# A Comparative Evaluation of Static Frictional Resistance Using Various Methods of Ligation at Different Time Intervals: An In Vitro Study

**DOI:** 10.1155/2015/407361

**Published:** 2015-03-31

**Authors:** Amanpreet Singh Natt, Amandeep Kaur Sekhon, Sudhir Munjal, Rohit Duggal, Anup Holla, Prahlad Gupta, Piyush Gandhi, Sahil Sarin

**Affiliations:** Dasmesh Institute of Research and Dental Sciences, Faridkot, Punjab, India

## Abstract

*Aim*. To compare and evaluate the static frictional resistance offered by the four different types of ligation methods in both dry and wet conditions and at different durations when immersed in artificial saliva. *Material and Methods*. Alastik Easy to Tie modules, Super Slick Mini Stix elastomeric modules, Power “O” modules, and 0.009^″^ Stainless Steel ligatures were used to compare the static friction using maxillary canine and premolar Preadjusted Edgewise brackets with 0.022^″^ × 0.028^″^ slot and 0.019^″^ × 0.025^″^ stainless steel wires. *Results*. The mean frictional resistance for Alastik modules was the lowest and that of Stainless Steel ligatures was found to be highest among the four groups compared and the difference among the four groups was statistically significant (*P* < 0.005). The mean static frictional resistance in all groups under dry conditions was lower than that under wet conditions. No statistical significant differences were found when the groups were compared at different time periods of immersion in artificial saliva. *Conclusion*. This study concludes that the Alastik modules showed the lowest mean static frictional forces compared to any other ligation method, though no significant difference was found for different time periods of immersion in the artificial saliva.

## 1. Introduction

The success of the straight wire appliance depends on the ability of orthodontic arch wire to slide freely through brackets and tube. During orthodontic tooth movement with sliding mechanics, a frictional force generated at the bracket/arch wire interface tends to impede the desired movement [1]. In clinical terms, the force applied must overcome this unknown frictional component and achieve the desired tooth movement.

Friction is an important factor in all forms of sliding mechanics such as space closure and canine retraction into an extraction site and in leveling and alignment where the wire must slide through the brackets and tubes. The nature of friction in orthodontics is multifactorial, derived from a multitude of both mechanical and biological factors. The method of ligation is an important contributor to the frictional force generated at the bracket/archwire interface. The new super slick modules introduced by TP Orthodontics and Alastik modules introduced by 3M Unitek claim to reduce friction more than other ligation methods [2]. Magnitude of friction depends upon the amount of normal force pushing the two surfaces together which is decided by the method of ligation, the surface roughness, and the nature of materials from which the surfaces are made [3].

The dissipation of the orthodontic force due to resistance to sliding may vary from 12% to 60%. On the other hand, an excessive increase in orthodontic force to overcome frictional resistance of the anterior teeth may produce increased posterior anchorage loss [4]. Baker et al. [5] using an artificial saliva substitute stated that 15 to 19% reduction in friction was seen in wet state, while some of the other studies [6, 7] showed that the coefficient of friction in the wet state is increased. Saliva could have lubricous as well as adhesive behavior, depending on which archwire bracket combination was under consideration.

So the aim of the present in vitro study was to compare and evaluate the frictional resistance offered by the Alastik Easy to Tie modules, Super Slick Mini Stix elastomeric modules, Power “O” modules, and 0.009′′ Stainless Steel ligatures for free sliding of stainless steel arch wires in stainless steel bracket slot in both dry and wet conditions.

## 2. Materials and Methods

The setup included the maxillary right canine and premolar Preadjusted Edgewise brackets with 0.022′′ × 0.028′′ slot and was of Roth Prescription (Ormco Corporation, Orange, CA). 0.019′′ × 0.025′′ stainless steel wires (Libral Traders, New Delhi, India) of 5 cm length were used to test friction during sliding movement in the bracket slots. In order to test the friction in wet state, the artificial saliva was prepared at Rajiv Academy of Pharmacy, Mathura, as described by Fusayama Meyer [8].

**Figure 1:**
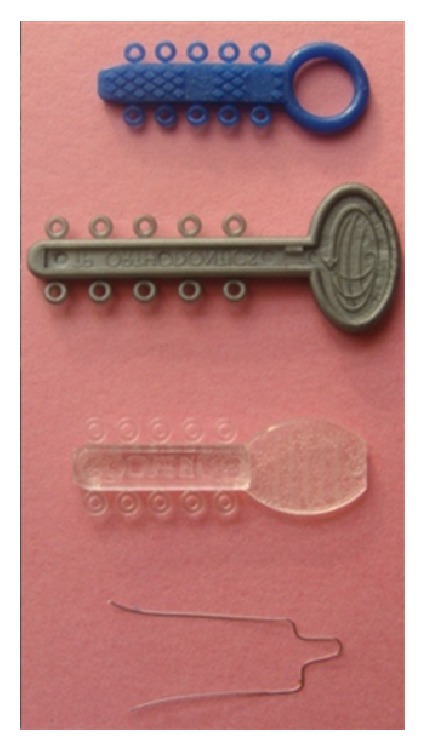
Four types of ligation materials used in the study.

Four types of ligation materials (Figure 1) used in the present study for ligating the wire to the bracket slots are mentioned as follows:

- GROUP I: Alastik Easy to Tie modules (3M Unitek, Minnesota, USA),
- GROUP II: Super Slick Mini Stix modules (TP Orthodontic, LaPorte, Indiana, USA),
- GROUP III: Power “O” modules (Ormco Corporation, Orange, CA),
- GROUP IV: Stainless Steel ligature, 0.009′′ (Libral Traders, New Delhi, India).

## 3. Sample

**Figure 2:**
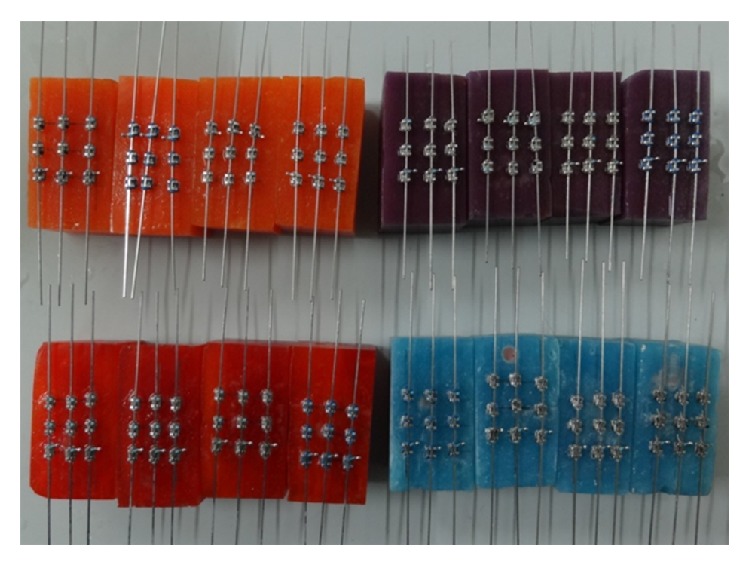
All custom made assemblies with different color coding of acrylic blocks.

**Figure 3:**
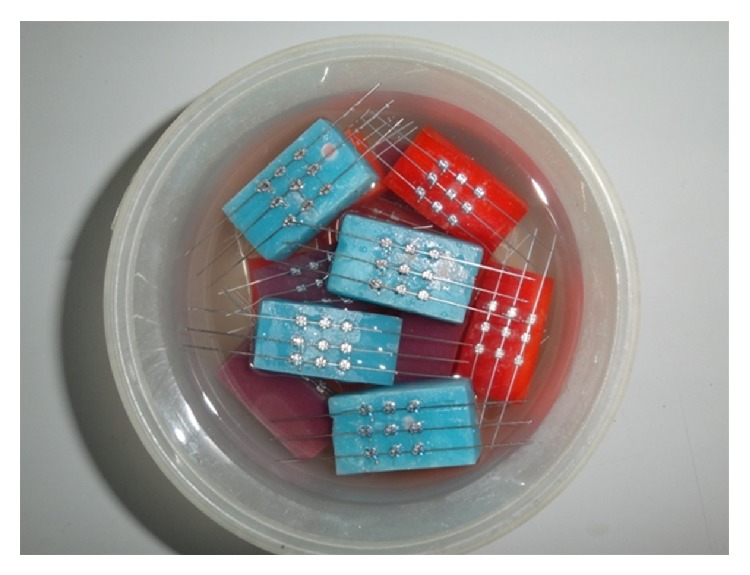
Custom made assemblies immersed in artificial saliva.

156 stainless steel right maxillary canine and premolar PAE brackets (39 brackets for each group) were used. Sample size consisted of 5 bracket-wire-ligation assemblies, each assembly consisting of 3 bracket-wire-ligation setups for each of the four groups tested (Figure 2). Each assembly was soaked in artificial saliva for 1 hour, 24 hours, 15 days, and 1 month before the test run and one sample was tested in dry condition (Figure 3). The acrylic blocks of different groups were color coded as per time intervals as described in the following:

 immediate (dry): white color;
 1 hour: blue color;
 24 hours: orange color;
 15 days: violent color;
 1 month: red color.

As 3 test readings were taken in each group for a particular time period, a total of sixty test runs were performed on the universal testing machine.

## 4. Methodology

**Figure 4:**
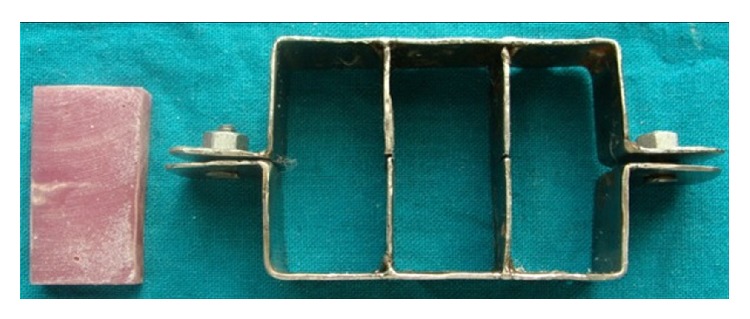
Metal housing in which acrylic block is fabricated (2′′ × 4′′).

A custom-made assembly was fabricated which consisted of acrylic block (2′′ × 4′′) prepared in a metal housing of the same dimensions (Figure 4). Once set, the acrylic block was removed and three sets of brackets-arch wire-ligation setup were bonded onto the acrylic block using an instant adhesive (Fevi Kwik, Pidilite Industries Ltd., Mumbai, India). Three right maxillary PAE brackets (canine, 1st premolar, and 2nd premolar) were bonded at 8 mm intervals and 5 cm long stainless steel straight length 0.019′′ × 0.025′′ wire was secured with the desired mode for ligation. This wire dimension was chosen because it is the recommended size for sliding mechanics with 0.022′′ slot brackets which were used in the present study.

**Figure 5:**
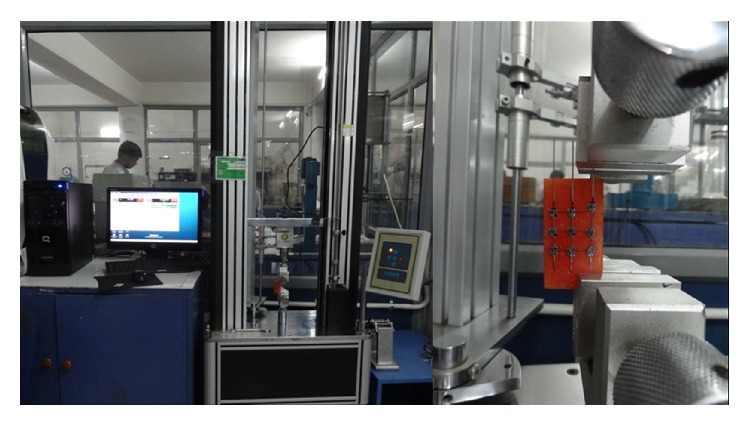
Custom made assembly mounted on the machine; test wire is being pulled in upward direction (universal testing machine, Blue Star, model number HZ 1004).

The wires were secured to test brackets with the elastomeric modules and the preformed ligatures (prepared with ligature forming plier) using artery forceps. The Stainless Steel ligatures were fully tied with the wire. The static friction between bracket and arch wire was measured with a universal testing machine (Blue Star Testing Service, India, Figure 5), with a crosshead speed of 20 mm/min as done by Hain et al. [2, 11] and Chimenti et al. [10]. The lower end of the assembly was attached to the lower crosshead of the testing machine. The wire was pulled in a vertical direction by the upper crosshead of the machine (Figure 5) till the 5 mm span of the wire was completely pulled out through the brackets. The force to overcome resistance to initiate movement of the wire was measured. This maximum frictional force at initial movement was taken to represent the peak static frictional resistance.

Values for peak static frictional forces (in grams) were recorded for each test run, from the electronic monitor display in the universal testing machine (Blue Star, model number HZ 1004) for each assembly.

## 5. Statistical Analysis

All the statistical tests were carried out with the SPSS software (IBM, Version 17, USA) using ANOVA and post hoc Tukey's test. Analysis of variance (ANOVA) was used to determine the variance in between the different groups with different ligation methods. A post hoc Tukey's test was then done to compare each type of ligation with respect to the others in each group and at different time periods. The power of the study was kept at .80 and the error of the study is 5%.

## 6. Results

Table 1 shows the mean static frictional resistance values with the standard deviations in each of the four groups at different time intervals, which were calculated using the one-way analysis of variance (ANOVA). It was seen that the mean static frictional resistance in all groups was lower in dry conditions sample as compared to wet conditions in all the time intervals. There were no statistical significant differences among four groups at the different time periods of immersion in the artificial saliva.

The mean static frictional resistance for Group I (Alastik modules) was the lowest and that of Group IV (Stainless Steel ligatures) was the highest among the four groups compared in wet conditions and the difference in the frictional resistance offered by the four groups was statistically significant (*P* < 0.005). Thus these results showed clear differences between the frictional resistance values generated with the different ligation methods.

A post hoc Tukey's test revealed that the Alastik and Super Slick modules showed statistically significant differences from the other ligation methods. The Alastik modules lowered the frictional resistance to an even greater extent than the Super Slick modules, Power “O” modules, and Stainless Steel ligatures. The coated Super Slick modules showed significantly lowered frictional resistance than the Power “O” modules and Stainless Steel ligatures.  Table 2 showed that the significant differences were found in the friction values between the immediate and 24-hour time periods in case of Group I (Alastik modules). The Group II (Super Slick modules) showed that no significant difference was seen in the different time periods of immersion.  Table 3 showed the significant difference in the frictional values between the immediate and 1-month time periods in case of Group III (Power “O” modules). These statics showed significant differences for immediate sample in all groups except in Group II (Super Slick modules) and Group III (Power “O” modules) which showed a nonsignificant difference in values.

**Figure 6:**
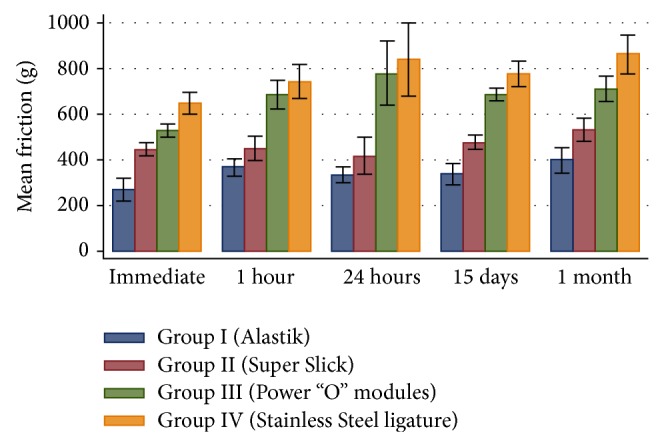
The bar diagram showing the mean static friction with standard deviations between the groups at different time periods (ANOVA test).

**Figure 7:**
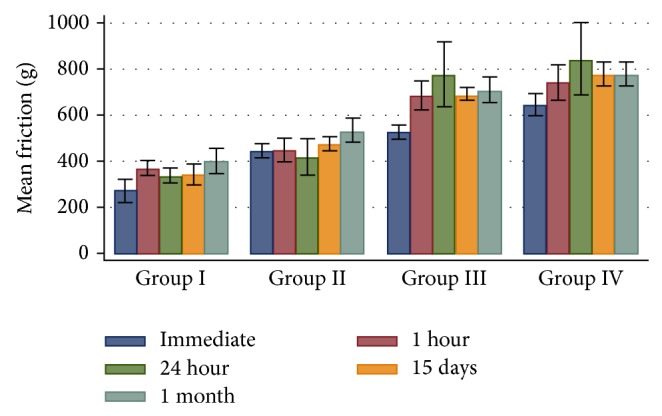
The bar diagram showing the mean static friction with standard deviations within the groups at different time periods (ANOVA test).

The bar diagram in Figure 6 shows the mean static friction with standard deviations between the groups at different time periods using ANOVA test and the bar diagram in Figure 7 depicts the mean static friction with standard deviations within the groups at different time periods.

## 7. Discussion

Many factors are involved within the bracket-arch wire-ligature system, which could influence the development of friction during sliding mechanics. This study was designed with the aim of standardizing as many of these factors as possible, so that the effects of different types of ligation methods could be objectively determined. Static friction was studied rather than the kinetic friction, since orthodontic tooth movement consists of a series of tipping and uprighting movements [4]. It is to be remembered that force required for overcoming static friction is greater than the force needed to sustain uniform sliding motion.

The results of this study showed that the 45° angulated Alastik Easy to Tie modules produced the lowest mean static friction. This result compares favourably with the results of Khambay et al. [9, 13] and Arun and Vaz [14] who found that Alastik Easy to Tie modules had the lowest mean frictional forces. A possible explanation could be that the bend in the module may prevent the entire module from contacting the wire. Even though the Alastik module seats the wire firmly into the bracket slot, the incomplete contact between the module and the wire may allow easier sliding [9], but the results of this study were in contrast to the study by Hain et al. [11] who found that Super Slick Mini Stix modules had lower mean frictional values than Alastik modules.

The results of present study also revealed that the Super Slick Mini Stix modules also exhibited lower mean frictional forces than the Power “O” modules and Stainless Steel ligatures but higher than the Alastik modules. This compares favourably with the studies of Griffith et al. [1], Khambay et al. [9, 13], and Arun and Vaz [14]. But the results are in contrast with the study of Hain et al. [2, 11] who found that Super Slick Mini Stix modules have low frictional values compared to Alastik modules. This was attributed to the presence of highly lubricious polymer coating, based on Metafasix technology, wherein a water insoluble coating has been covalently bonded to the ligatures, E-chains, and separators causing a slippery surface when moistened reducing the friction as much by 70% as claimed by the manufacturer. Preangulated Alastik modules showed lower mean frictional force when compared with the Super Slick modules, though the difference was statistically not significant, implying that both the elastomeric ligatures were equally efficient in reducing frictional resistance when compared with Power “O” modules and Stainless Steel ligatures.

When comparing the results of Power “O” modules, it showed lower mean frictional force than the stainless steel ligatures but higher than the Alastik modules and Super Slick Mini Stix modules. These results compare favourably with the results of Griffith et al. [1], Hain et al. [11], Gandini et al. [12], and Arun and Vaz [14] but are in contrast with the results of the Khambay et al. [9, 13] who showed that regular modules have low frictional forces compared to the Super Slick Mini Stix modules because these modules need to remove the saliva film as the wire translates beneath them during sliding.

It is seen that the Stainless Steel ligatures had higher mean frictional force than the Alastik, Super Slick Mini Stix, and Power “O” modules. These results are in contrast with the studies of Hain et al. [2] and Khambay et al. [9, 13] who showed the lowest mean frictional force by the stainless steel ligature compared to any other ligation method. A possible explanation for this might be that in our study we fully ligated the Stainless Steel ligature with the wire but Khambay et al. [9, 13] and Bazakidou et al. [15] gave seven full turns of Spencer-Wells clips/Mathieu ligature tying plier after the ligature was placed and was ready for tightening. Rajendran et al. [8] in their study initially fully tightened the ligature and then unwound it by 3 turns, but we found that unwinding the ligature by 3 turns would make the ligature very loose. In the present study, the value of frictional resistance offered by the stainless steel ligature was high as it may have been tighter than that done in other studies.

The effect of lubrication is debatable and the increased or decreased frictional resistance cannot be attributed to the lubricant used with any certainty [16, 17]. This study indicates that, with all types of the ligation materials, the static friction was increased in the wet conditions relative to the dry conditions. The results compare favourably with the studies of Edwards et al. [18], Griffith et al. [1], Stannard et al. [7], and Thorstenson and Kusy [19]. It could be due to the fact that these modules have to remove the saliva film as the wire translates beneath them during sliding.

Though it is not possible to reproduce and standardize the exact oral environmental conditions that influence the friction clinically, an attempt has been made to test friction at different time periods immersed artificial saliva with different ligation methods. The study shows that the saliva immersion increased the friction in wet conditions as compared to the dry condition but no significant difference was seen in each group between different immersion periods.

Difficulty in comparing the results of this study with the previous studies of the different elastomeric modules could be due to the differences in the methodologies and Table 4 shows the comparison of methodologies between the present and the previous studies. However, more research is needed to enhance our understanding of different methods of ligation and its effects on various bracket systems.

## 8. Summary and Conclusions

(1) The Alastik Easy to Tie modules showed the lowest mean static frictional forces compared to any other ligation method. This finding could be attributed to the bend in the module which prevents the entire module contacting the wire.

(2) The Super Slick Mini Stix elastomeric modules showed lower mean static frictional forces than the Power “O” modules and Stainless Steel ligatures but higher than the Alastik Easy to Tie modules. Though there was no statistically significant difference between the two. This finding could be attributed to the surface characteristics of these modules.

(3) The Power “O” modules showed lower mean static frictional forces than the Stainless Steel ligatures but higher than the Alastik Easy to Tie modules and Super Slick Mini Stix modules.

(4) The Stainless Steel ligatures showed the highest mean static frictional forces compared to any other ligation method. This finding could be attributed to the tight ligation by these ligatures with the wire.

(5) This study showed that, with all four types of the ligation methods, the static friction was increased in the wet conditions relative to the dry condition, though no significant difference was found for the static frictional resistance for different time periods of immersion in the artificial saliva.

## Figures and Tables

**Table 1:** Comparison of mean static friction between different groups (ANOVA test).

	Time	Mean	Standard deviation	*F*-ratio	*P* value	NS/S
GP I	Immediate (dry)	270.00	50.00000	3.497	**0.049**	**S**
	1 hour	370.00	36.05551			
	24 hours	336.67	32.14550			
	15 days	340.00	45.82576			
	1 month	400.00	55.67764			

GP II	Immediate (dry)	443.33	30.55050	2.060	0.161	NS
	1 hour	450.00	52.91503			
	24 hours	420.00	79.37254			
	15 days	476.67	32.14550			
	1 month	533.33	51.31601			

GP III	Immediate (dry)	526.67	30.55050	4.488	**0.025**	**S**
	1 hour	686.67	65.06407			
	24 hours	780.00	141.06736			
	15 days	690.00	26.45751			
	1 month	710.00	30.55050			

GP IV	Immediate (dry)	646.67	45.09250	2.605	0.100	NS
	1 hour	743.33	65.06407			
	24 hours	843.33	159.47832			
	15 days	780.00	26.45751			
	1 month	863.33	55.67764			

**Table 2:** Post hoc Tukey's test for Group I (Alastik).

Time	Immediate (dry)	1 hour	24 hours	15 days	1 month
Immediate (dry)	—	NS	**S**	NS	NS
1 hour	NS	—	NS	NS	NS
24 hours	**S**	NS	—	NS	NS
15 days	NS	NS	NS	—	NS
1 month	NS	NS	NS	NS	—

S: significant difference (*P* < 0.05); NS: nonsignificant difference.

**Table 3:** Post hoc Tukey's test for Group III (Power “O” modules).

Time	Immediate (dry)	1 hour	24 hours	15 days	1 month
Immediate (dry)	—	NS	NS	NS	**S**
1 hour	NS	—	NS	NS	NS
24 hours	NS	NS	—	NS	NS
15 days	NS	NS	NS	—	NS
1 month	**S**	NS	NS	NS	—

S: significant difference (*P* < 0.05); NS: nonsignificant difference.

**Table 4:** Comparison of mean static friction found in other studies quoted and results obtained in the present study. A comparative evaluation of static frictional resistance.

Author	Type of bracket-wire used in the study and method of ligature placement	Type of ligation	Dry or wet medium	Mean static friction	
Stannard et al. [7], 1986	Stainless steel or Teflon coated brackets with 0.017 × 0.025′′ SS, TMA, NiTi, and Co-Cr wires Placement of ligature not mentioned	Stainless Steel ligatures	Both	*Dry* 739 g SS	*Wet* 855 g SS

Baker et al. [5], 1987	PAE SS Brackets with 0.018′′, 0.020′′, and 0.018 × 0.025′′ SS wires Placement of ligature not mentioned	0.010′′ polyurethane ligatures	Both	142 g in saliva 170 g in dry 166 g in glycerine	

Dowling et al., 1998	Standard twin and mini twin brackets with 0.018 × 0.025′′ wire Placement of ligature with an Orthopli 018R forceps	Elastomeric modules round A-grey, B-clear, C-orange, D-fluoride impregnated, rectangular E-grey	Both	*Dry* A-1.05 N B-1.06 N C-0.91 N D-1.16 N E-1.46 N	*Wet* A-1.25 N B-0.80 N C-1.22 N D-1.07 N E- 1.15 N

Khambay et al. [9], 2005	Self-ligating Damon II and PAE SS brackets with 0.017 × 0.025 SS and TMA and 0.019 × 0.025′′ SS and TMA Placement of ligature with straight shooter gun	Elastomeric modules-, purple, grey, Alastik or Super Slick, and 0.09′′ SS ligature	Wet	*0.019 × 0.025*′′* bracket* SS ligature-0.45 N Alastik-0.50 N Purple-0.56 N Grey-0.84 N Super Slick-0.98 N	

Chimenti et al. [10], 2005	0.022′′ PAE SS brackets with 0.019 × 0.025′′ SS wire Placement of ligature not mentioned	Elastic modules (small, medium, large, clear lubricated, and grey lubricated)	Dry	Small modules: 533.16 g Medium modules: 508.80 g Large modules: 611.14 g Clear lubricated: 392.44 g Gray lubricated: 350.38 g	

Hain et al. [11], 2006	Victory, speed, and Damon II brackets with 0.019 × 0.025′′ SS wire Placement of ligature not mentioned	Regular uncoated, Super Slick, conventional silver, Alastik, Sili-Ties	Wet	Regular uncoated: 2 N Super Slick: 0.96 N Conventional silver: 2.80 N Alastik: 1.87 N Sili-Ties: 1.81 N	

Gandini et al. [12], 2008	SmartClip and conventional SS bracket with 0.014′′ NiTi and 0.019 × 0.025′′ SS wire Placement of ligature not mentioned	Conventional elastomeric ligature (CEL) and unconventional elastomeric ligature (UEL)	Dry	*0.019 × 0.025*′′* SS* CEL-177.4 g UEL-1.2 g	

Present study, 2014	0.022′′ PAE SS bracket with 0.019 × 0.025′′ SS wire Placement of ligature with artery forceps	Grp. I: Alastik Easy to Tie Grp. II: Super Slick Mini Stix Grp. IIII: Power “O” modules Grp. IV: 0.009′′ Stainless Steel ligature	Both dry and wet mediums at different time intervals	*Dry* Grp. I: 270 g Grp. II: 443 g Grp. III: 526 g Grp. IV: 646 g	*Wet* Grp. I: 343 g Grp. II: 464 g Grp. III: 678 g Grp. IV: 775 g
